# Aerobiology and Environmental Zonation in Gypsum Caves: A Comparative Study of Culturing and NGS Approaches

**DOI:** 10.1007/s00248-025-02591-4

**Published:** 2025-09-30

**Authors:** Tamara Martin-Pozas, Angel Fernandez-Cortes, Jose Maria Calaforra, Sergio Sanchez-Moral, Cesareo Saiz-Jimenez, Valme Jurado

**Affiliations:** 1 Instituto de Recursos Naturales y Agrobiologia de Sevilla, IRNAS-CSIC, 41012 Seville, Spain; 2 Departamento de Biologia y Geologia, Universidad de Almeria, 04120 Almeria, Spain; 3 Museo Nacional de Ciencias Naturales, MNCN-CSIC, 28006 Madrid, Spain

**Keywords:** Aerobiology, Cave aerobiome, Cave environment, NGS, SAS, Bacteria

## Abstract

Classical aerobiological studies commonly use high-volume air samplers to quantify and identify cultivable airborne bacteria and fungi. However, this approach introduces a significant bias, as it overlooks the non-cultivable fraction, which likely constitutes a major component of the airborne microbiome. The advent of next-generation sequencing (NGS) has addressed this limitation, enabling a more comprehensive characterization of the cave aerobiome. This study analyzes both cultivable and non-cultivable airborne bacteria from Covadura and C3 caves, located in the Gypsum Karst of Sorbas (SE Spain). A total of 24 bacterial genera were identified using culture-based methods, whereas NGS revealed 749 genera. Culture-based methods using the surface air system (SAS) predominantly recovered Gram-positive spore-forming bacteria from the phyla Bacillota and Actinomycetota, which were largely absent or present in low relative abundances in the NGS datasets. In contrast, NGS revealed a broader diversity, including numerous Gram-negative and rare airborne bacteria not detected by culture. The NGS results from airborne samples showed greater similarity to the microbial communities found in cave biofilms and sediments, suggesting that a portion of airborne bacteria originates from within the cave and is influenced by microclimatic conditions such as ventilation and air stagnation. Although the short-read sequencing approach used in this study has limitations, such as reduced taxonomic resolution compared to the culture-based approach, it remains the most effective tool for capturing the diversity and ecological patterns of airborne microorganisms. The integration of gas tracers and other environmental data allowed the identification of zones within the cave with different ventilation patterns and degrees of isolation, which corresponded to different spatial distributions of airborne bacteria. Our findings underscore that reliable aerobiological studies in caves require the combination of non-culture dependent-based sequencing approaches and environmental monitoring to fully understand the origin, diversity, and ecological dynamics of airborne microbial communities.

**Supplementary Information:**

The online version contains supplementary material available at 10.1007/s00248-025-02591-4.

## Introduction

Geomicrobiology has advanced significantly in the last decades. Studies on cave microbial communities have been driven by different reasons, among others, the need to avoid microorganism outbreaks in show caves and the isolation and description of new species of cave bacteria and fungi, which were discovered to be sources of compounds of biotechnological interest [1, 2].

Caves are inhabited by microorganisms, including bacteria, fungi, algae, and protozoa, as well as different animals. Microorganisms are found on the rock walls, speleothems, sediments, drip and stagnant waters, as well as in the air. The transport of microorganisms from the exterior into the cave follows different ways: air, infiltration waters, and visits. All these ways contribute decisively to their distribution across the caves [3, 4].

Aerobiology was founded on the studies of pollens and fungi and their involvement in allergic processes [5]. Perhaps one of the least investigated areas in caves is aerobiology, the study of biological particles transported and dispersed by the air. Comparatively, cave aerobiological studies are less numerous than those focused on rock, sediment, and water microbiomes [6].

In recent years, only a few limestone caves in Spain have been subjected to aerobiological studies [3, 7, 8]. These investigations were carried out with large-volume collectors and investigated the dispersion of bacteria and fungi along the cave galleries and their relationship with other microbial communities present in the sediments and wall biofilms. However, these studies were carried out on microorganisms that can be easily cultivable in the laboratory, providing incomplete information on the total population present in cave air. However, the fraction of non-cultivable microorganisms remained unknown. In general, in the cultivable fraction of limestone caves, the occurrence of the same bacterial genera and species is a common trend and provides little information on the cave aerobiome [7, 8].

In 2007, Saiz-Jimenez and Gonzalez [9] stated that “In the near future, modern molecular tools will considerably expand our knowledge of the diversity of airborne microorganisms by enabling the detection of a wide range of so far uncultured microorganisms. Implementing these techniques into classical sampling procedures used in aerobiology will be one of the first requirements to be met.” In recent years, methods based on sequencing of the 16S rRNA gene of bacteria and the ITS region of fungi that do not require microorganism’s cultivation have been used. These include studies on bioaerosols [10, 11], cultural heritage [12], atmosphere and stratosphere [13], among others. Several authors have reviewed the progress of aerobiology in recent years and have emphasized the development of omics techniques and, particularly, NGS (next-generation sequencing) technologies for the study of environmental DNA (eDNA) [14–16].

To fully understand the dynamics of airborne microbial communities in caves, it is essential to integrate aerobiological data with environmental variables, such as temperature, humidity, airflows, and trace gases, such as CO_2_ and CH_4_ concentration. Environmental conditions in subterranean settings are known to strongly influence microbial dispersal, survival, and colonization. For instance, air circulation patterns can act as vectors for microbial transport and determine the spatial heterogeneity of airborne microbiota and biofilm development [17]. This integrative approach was emphasized by Sanchez-Moral et al. [18], who demonstrated that the structures of the fungal community in show caves were strongly controlled by environmental conditions such as relative humidity and ventilation regimes. Similar conclusions have been reached in other cave environments, where the interaction between physical cave parameters and microbial occurrence has been shown to be crucial for interpreting both microbial diversity and potential ecological impacts. Combining aerobiological studies with high-resolution environmental monitoring can thus provide a more comprehensive picture of cave microbial ecology, particularly in the context of human visitation and climate-driven changes in cave microclimates.

Previous aerobiological studies in Spain focused on limestone caves, but gypsum caves have not been studied until now, and it is unknown whether the bacterial genera and species in gypsum caves are the same as those usually collected in limestone caves. This work focuses on the cultivable and non-cultivable fractions of bacteria from two caves, Covadura and C3, in the Gypsum Karst of Sorbas (Almeria, Spain), and the data obtained from the two different approaches are compared and discussed. In addition, environmental parameters such as temperature, relative humidity, and CO_2_ and CH_4_ levels were monitored during the sampling campaigns to determine how, and to what extent, these factors shape the dispersion of airborne microorganisms within the two caves studied.

## Material and Methods

### Study Site

**Fig. 1:**
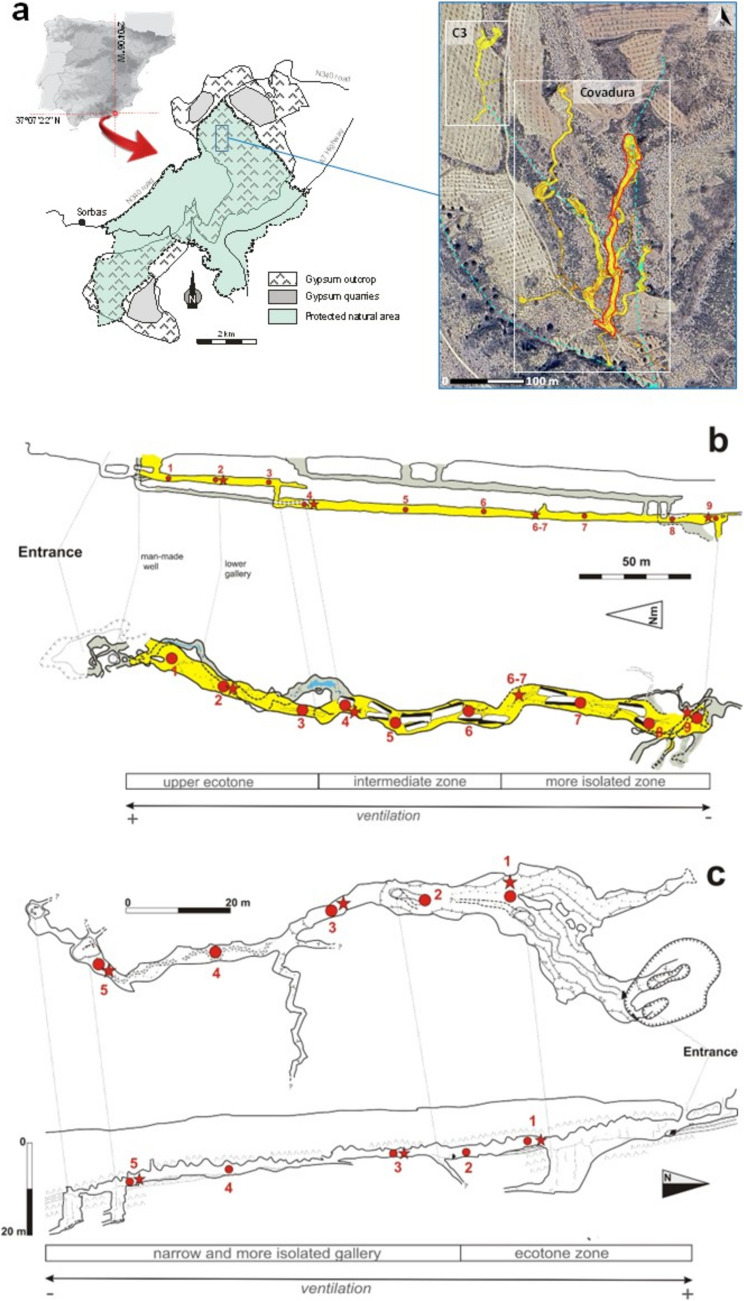
**a** Location of the Gypsum Karst of Sorbas and spatial distribution of the galleries of Covadura and C3 caves (yellow areas) in relation to the external topography (modified from Jurado et al. [50]. The dashed blue lines show the drainage network on the surface. The yellow areas highlighted by a red line correspond to the galleries studied in Covadura Cave. **b**, **c** Network of microclimate and air sampling points (red squares) and aerobiological sampling stations (red starts) distributed along Covadura Cave (**b**) and C3 Cave (**c**). The yellow areas in Covadura Cave correspond to the galleries sampled in this study, in relation to the rest of the surrounding galleries. The main cave sectors are distinguished in terms of the aerodynamic connection with the exterior and are based on the microclimate data (see text). Cave maps (floor plans and profiles) modified from Ayuso et al. [21]

The Gypsum Karst of Sorbas (37°05′31″ N, 2°06′27″ W) is located in the semi-arid region of southeast Spain (Fig. 1), characterized by an average annual temperature of approximately 19.5 °C, with a minimum average of 11 °C in January and a maximum of 30 °C in July [19]. The mean annual precipitation is about 210 mm, with a common dry summer and the highest rainfalls recorded during autumn.

The karst comprises many caverns. Covadura Cave is one of the longest (4.25 km of galleries distributed over six depth levels) and deepest (126 m) gypsum caves in Spain [20]. C3 Cave is located 200 m from the western access to Covadura. This cave is a blind gallery, 3 m deep, and has a length of 150 m, which becomes part of the upper levels of the Covadura system. This gallery ends in a narrow shaft with a probable hydric connection with other galleries in the system.

The topography of both caves, with a main entrance located at a higher level than the rest of the cave, determines a spatial air temperature pattern characterized by a decrease from the entrance of the cave to the interior. Previous studies in Covadura reported a prevailing steeper temperature gradient (cooling with depth) throughout the year and an increase in spatiotemporal thermal stability of the air in the deeper galleries, with an average temperature of the cave air ranging from 9.2 to 14.6 °C [21]. The highest thermal stability was identified beyond 90 m from the main entrance. The highest air temperatures were recorded in September and the coldest during February [19]. During the colder period, from October to May, when the temperature of the external air is usually lower than that of the cave air, the external cool air tends to enter the upper galleries, and the warmer cave air is partially evacuated.

### Aerobiological Sampling

Aerobiological samplings are usually carried out in different seasons of the year, or at least in winter and summer. This has not been possible due to the restrictions imposed by the Environment Department of the Andalusian Government and the prohibition of access to the caves for a long period, including the hibernation of bats inhabiting the caves. Therefore, data from a single sampling in Covadura and C3 caves are presented, but the results allowed for a comparison between the two methodological approaches.

Eighteen air samples in total were taken from both inside and outside Covadura and C3 caves at the same time with two different air samplers in a campaign carried out on October 4, 2023, (C3 Cave) and October 5, 2023 (Covadura Cave). The sampling points are detailed in Table S1 and indicated on the cave map (Fig. 1). Figure S1 compiles a representative set of pictures of the cave galleries and the dolines to access the upper levels of Covadura and the main gallery of C3 cave. The samples include locations near the cave entrance, interior galleries, as well as outside control. As this was an exploratory study, no biological replicates were included for the NGS analyses. For culture data, two replicates were included. The primary objective was to compare general trends between sampling methods and sites.

### Monitoring of Microclimate and Cave Air Renewal

Cave air monitoring was conducted to obtain key data for understanding the current cave environmental conditions during the two aerobiological samplings mentioned above, including air temperature, relative humidity, and the gaseous composition of cave air, external soil, and local background atmosphere.

Grab air samples were collected with a portable air compressor (0.4 L·min^−1^), and soil air was extracted using a microdiaphragm gas pump (4.5 L·min^−1^). Air samples were stored in 1-L RITTER gas bags and analyzed in 48 h for CO_2_ and CH_4_ molar fractions and δ^13^C-CO_2_ and δ^13^C-CH_4_ values using a G2201-i CRDS analyzer (Picarro, USA). Certified standards were used to verify instrument performance. Further methodological details are provided in [22].

Air temperature and relative humidity were also taken at the same cave locations as air samples using a handheld XP200 monitor (Lufft, Germany), equipped with an external PT100 1/10 DINB probe for temperature (accuracy of ± [0.03 + 0.002 ∗ measurement]) and a capacity probe for relative humidity (measurement range: 0–100%, accuracy: ± 3% above 90%). All devices had certified calibrations.

### Cultivable Bacteria: Isolation and Identification

A high-volume sampler surface air system (SAS) DUO, SUPER 360, PBI International, Milan, Italy, was used for the aerobiology of gypsum caves, which allows the identification of cultivable bacteria and fungi [7, 23]. The DUO SAS draws air through two heads provided with numerous holes. A Petri dish with culture medium is inserted into each head, on whose surface the air impacts, which will favor the growth of the cultivable microorganisms. The volume of air collected was 100 L and the culture medium used for the isolation of bacteria was trypticase soy agar (TSA, BD, Heidelberg, Germany). The Petri dishes were incubated at 28 °C for 5 days, after which the grown colonies were counted and the values were expressed as colony forming units per cubic meter of air (CFU/m^3^). Subsequently, the bacteria were isolated and cultured in TSA. The isolated bacteria were identified by Sanger sequencing of the 16S rRNA gene using the primers 616 F 5′-AGAGTTTGATYMTGGCTCAG-3′ [24] and 1510R 5′-GGTTACCTTGTTACGACTT-3′ [7]. The determination of the degree of similarity between the isolated bacteria and collection type species was carried out using the BLAST algorithm in the NCBI database (National Centre for Biotechnology Information, https://www.ncbi.nlm.nih.gov/). The EzBioCloud server was also used (https://www.ezbiocloud.net).

### Non-cultivable Bacteria: DNA Extraction and Sequencing

A Coriolis µ air sampler (Bertin Technologies, Montigny-le-Bretonneux, France) was used for non-cultivable microbial analysis [4]. The Coriolis sampler collected a total of 3000 L of air through a container with a solution (Triton X-1000 at 0.005%) in which airborne particles were retained. The samples were kept on ice until they arrived at the laboratory where they were directly processed. The air samples in Triton-X-100 were filtered using a 0.22 µm diameter filter (Millipore, Billerica, USA), and the DNA was extracted from the filter using the Fast DNA SPIN Kit for Soil (MP Biomedicals, Solon, USA) following the manufacturer’s instructions. DNA concentrations were quantified using a Qubit 2.0 fluorometer (Invitrogen, Carlsbad, USA). Negative controls were included throughout the entire protocol to identify potential contamination in the low-biomass air samples. DNA was not detected in the negative controls using the Qubit 2.0 fluorometer. The V3 and V4 regions of 16S ribosomal RNA were amplified using the primer sequences 341 F (CCTACGGGNGGCWGCAG) and 805R (GACTACHVGGGTATCTAATCC) [25], using Illumina MiSeq and 2 × 300 paired-end sequencing. NGS of the extracted DNA was performed in the company FISABIO (Valencia, Spain).

### Bioinformatics and Taxonomic Assignment

Sequence data were processed using Qiime2 pipeline [26]. Taxonomic analysis was performed using some of the Qiime2 plugins. Denoising, paired-end joining, and chimera removal were performed from the paired-end data using the DADA2 pipeline [27]. For quality filtering, reads were removed when more than 50% of the sequences had a Phred score below 20. A total of 954,495 high-quality reads from nine DNA samples (per-sample sequencing depth: min = 76,887; max = 150,502; mean = 106,055) were clustered into 12,861 ASVs. Taxonomic affiliations were assigned using the Naive Bayes classifier integrated in Qiime2 plugins. The database used for taxonomic assignment was SILVA SSU 138 [28]. Sequences identified as chloroplast and mitochondria were removed.

Data were imported to R environment for statistical analyses. Samples were rarefied to an even sequencing depth of the minimum sample depth. Alpha diversity was analyzed using Shannon–Wiener and Simpson indices to assess the diversity of samples. The Chao1 index was used to assess the species richness of the samples. ANOVA, Kruskal–Wallis, and post hoc analysis were used to compare the diversity indices across the different environments. Non-metric multidimensional scaling (NMDS) based on Bray–Curtis dissimilarities was done at the species level.

## Results

### Microclimate Conditions

Figure 1 shows the network of sampling stations in Covadura and C3 caves. Table 1 provides the results of the microclimatic parameters and gaseous composition of cave air across different subzones of the subterranean ecosystem.

**Table 1:** Temperature, relative humidity, and gaseous composition (CO_2_ and CH_4_) of the subterranean atmosphere of the Covadura and C3 caves (COV-# and C3-#, respectively)

Sample	T (°C)	RH (%)	CO_2_ (ppm)	δ^13^C-CO_2_ (‰)	CH_4_ (ppm)	δ^13^C-CH_4_ (‰)
**Exterior**	29.56	34.4	426	− 9.26	2.01	− 52.40
			424	− 9.42	2.01	− 51.22
	27.51	56.7	425	− 9.15	1.99	− 51.03
			422	− 9.48	2.00	− 51.46
**Exterior (avg.)**	**28.53**	**45.6**	**424**	** − 9.33**	**2.00**	** − 51.53**
**Soil**	-	-	1407	− 20.68	0.53	− 48.43
	-	-	1523	− 21.09	0.29	− 32.05
**Soil (avg.)**			**1465**	** − 20.89**	**0.41**	** − 40.24**
**C3-1**	16.60	89.3	588	− 13.22	0.36	-
**C3-2**	-	-	524	− 11.59	0.38	-
**C3-3**	17.83	88.9	611	− 11.25	0.21	-
**C3-4**	17.48	91.7	542	− 12.27	0.25	-
**C3-5**	17.19	92.1	533	− 12.15	0.22	-
**C3 (avg.)**	**17.28**	**90.5**	**560**	** − 12.09**	**0.28**	**-**
**COV-1**	16.29	78.9	431	− 9.47	1.94	− 50.94
**COV-2**	-	-	468	− 10.68	1.96	− 51.33
**COV-3**	19.66	89.0	440	− 9.89	1.98	− 52.05
**COV-4**	14.09	90.7	454	− 10.35	1.79	− 48.87
**COV-5**	13.73	92.8	462	− 10.80	1.78	− 49.96
**COV-6**	13.12	93.5	444	− 10.36	1.70	− 49.11
**COV-7**	12.05	93.3	460	− 10.80	1.44	− 47.33
**COV-8**	11.80	94.3	495	− 11.78	1.23	− 45.66
**COV-9**	12.09	95.1	521	− 12.54	1.19	− 48.93
**Covadura (avg.)**	**14.10**	**90.9**	**464**	** − 10.74**	**1.67**	** − 49.35**

**Fig. 2:**
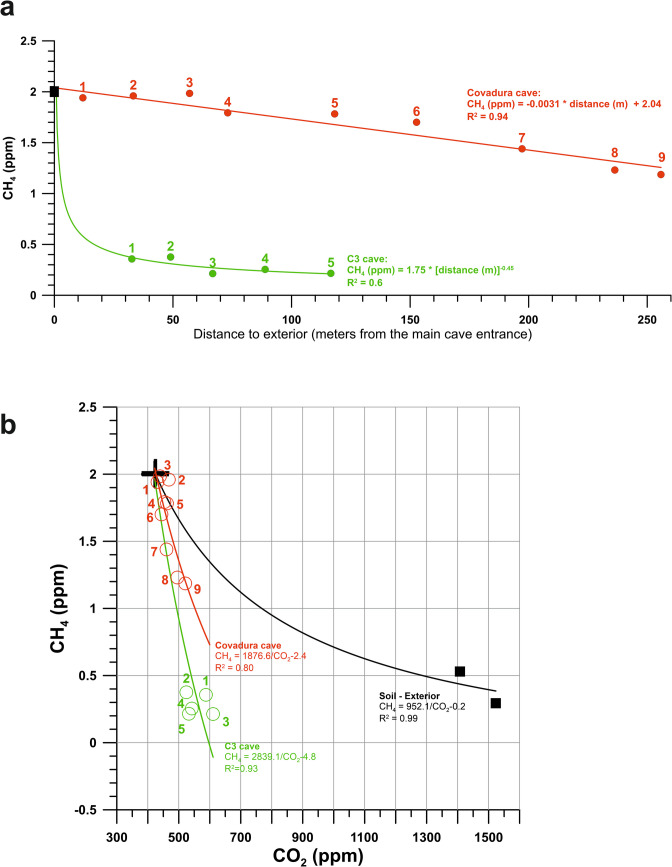
Gas concentrations along the cave-soil-atmosphere profile of Covadura and C3 caves. **a** Profiles of CH_4_ concentration of cave air in function of the distance to exterior. **b** Variations in tracer gas concentrations (CO_2_ and CH_4_) along the cave-soil-atmosphere profile of Covadura and C3 caves. Cave air (open circles, numbers-indicated labels correspond to cave locations in Fig. 1), soil (solid black squares) and black crosshairs (local atmosphere at exterior). The colored lines correspond to the fitted inverse functions (CH4 = k/CO2 + b) that are indicative of atmospheric CH_4_ due to the influx of outside air into the cave through advective processes, compared to the consumption of CH_4_ in the external soil layer. An increase in coefficients implies a higher depletion of CH_4_

The environmental parameters revealed distinct differences between the two cave systems. The profile of CH_4_ concentration as a function of the distance to the exterior (Fig. 2a) provides information on the spatial connection pattern of the cave galleries with the exterior atmosphere. C3 Cave exhibited a relatively high and stable temperature (mean: 17.28 °C) and relative humidity (mean: 90.5%). In contrast, Covadura Cave showed a cooler microclimate (mean temperature: 14.10 °C; mean relative humidity: 90.9%) than C3, with significantly lower temperatures than the exterior environment (28.53 °C).

The CO_2_ concentrations within C3 Cave were elevated compared to ambient atmospheric levels (mean: 560 ppm vs. external 424 ppm), while the CH_4_ concentrations were markedly depleted (mean: 0.28 ppm vs. external 2.00 ppm). The environmental conditions were homogeneous throughout the sampling area; however, slightly lower CH_4_ levels were observed in the deeper zone (from C3-3 to C3-5; Table 1) compared to the entrance-proximal subarea (C3-1 and C3-2; Table 1). On the contrary, Covadura Cave presented higher CH_4_ concentrations (mean: 1.67 ppm) and lower CO_2_ concentrations (mean: 464 ppm), both gases closer to atmospheric values.

Figure 2b shows the CH_4_ depletion coeval to increments of CO_2_ concentration in the underground air of Covadura and C3 caves. Air renewal during the aerobiology survey was markedly higher in Covadura Cave than in C3 Cave, although C3 Cave is closer to the surface and shorter in length, resulting in a higher rate of depletion of CH_4_ with respect to Covadura (CH_4_ < 0.5 ppm and CH_4_ > 1 ppm, respectively). Along Covadura Cave, the CH_4_ concentration and δ^13^C-CH_4_ isotopic signatures are close to atmospheric background levels (~ 1.9–2.0 ppm and − 51 to − 52‰) in the upper gallery near the surface. In the lower gallery, the values range from 1.7 to 1.8 ppm and − 49 to − 50‰, further decreasing to concentrations below 1.5 ppm and δ^13^C-CH_4_ values lower than − 49‰ in the deepest and most isolated sections of the cave, at 190 m from the main entrance (Table 1 and Fig. 2b). The results for Covadura Cave indicate a CH_4_ depletion rate of 0.08 ppm, on average, for each 10 ppm increase in CO_2_. Based on these results, three ventilation subzones were identified within Covadura Cave (Fig. 1a): (i) the innermost, less ventilated and coldest section (COV-1 to COV2-3); (ii) an intermediate zone (COV-3 to COV-4) with moderate air exchange; and (iii) the upper ecotone sector (COV-5 to COV-6), characterized by higher temperature and substantial air renewal.

### Cultivable Bacteria

**Fig. 3:**
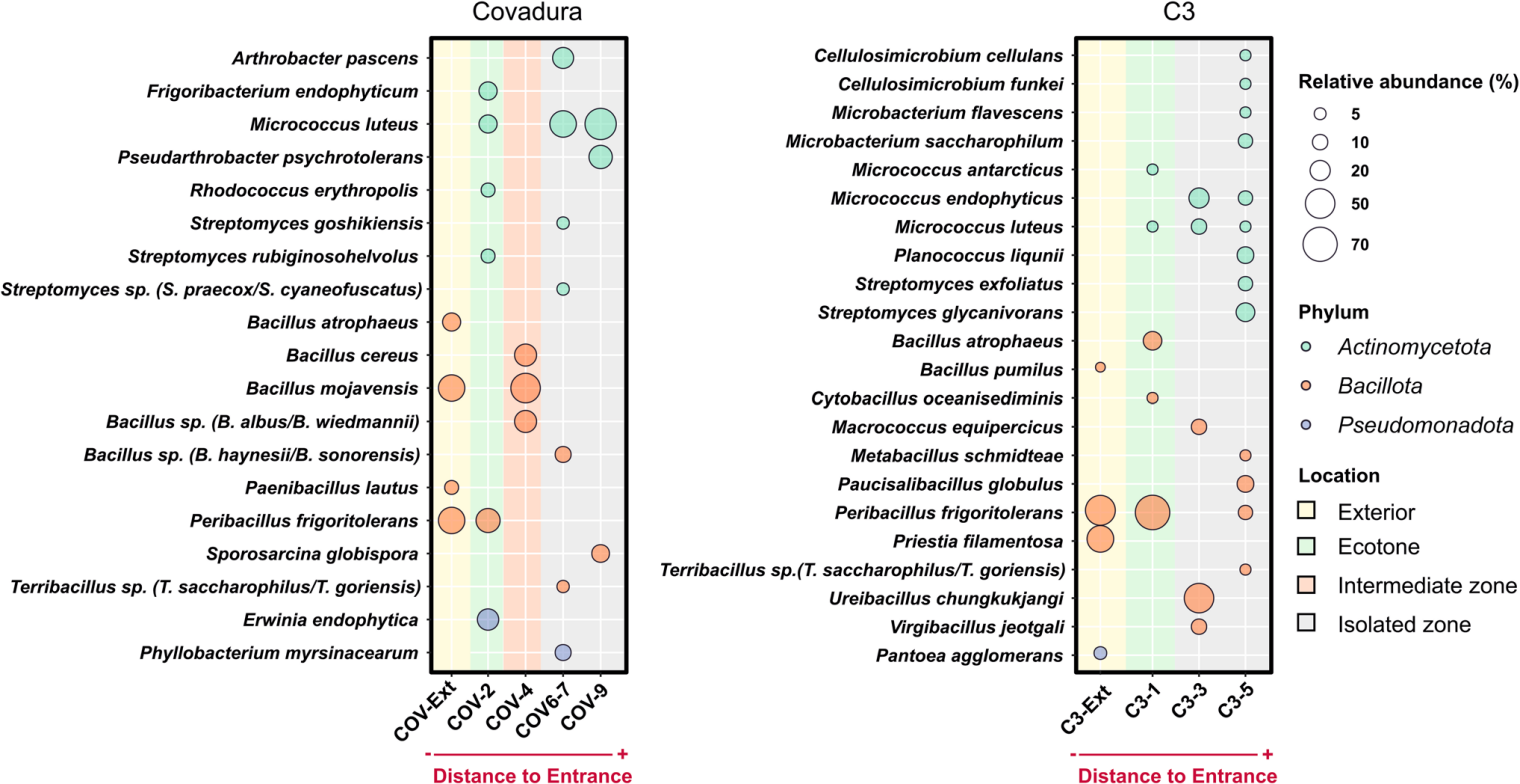
Bubble plot showing the relative abundance of cultivable bacteria in the air of Covadura Cave (left panel, (COV-#) and C3 Cave (right panel, C3-#). Bubble size represents the percentage of relative abundance. Circle colors indicate bacterial phyla, and square colors indicate location

Cultivable bacteria in Covadura and C3 caves are shown in Fig. 3. This methodology identified only 37 bacterial species. The galleries of Covadura Cave and the exterior air are notable for the abundant occurrence of cultivable genera and species of the Bacillota phylum, including *Bacillus*, *Terribacillus*, *Paenibacillus*, and *Peribacillus* (Table S2). This abundance in the galleries is usually accompanied by Actinomycetota, particularly *Micrococcus luteus*, *Pseudarthrobacter psychrotolerans*, and *Arthrobacter pascens*, and in lesser amounts by *Streptomyces* spp. and *Rhodococcus erythropolis*. Of interest is the abundant occurrence of *Peribacillus frigoritolerans* and *Bacillus mojavensis* inside and outside the cave.

In C3 Cave, Bacillota are abundantly represented, although with a greater diversity: *Bacillus*, *Peribacillus*, *Cytobacillus*, *Virgibacillus*, *Ureibacillus*, *Metabacillus*, *Terribacillus*, and *Paucisalibacillus* (Table S3). *Peribacillus frigoritolerans* is very abundant both outside and inside the cave, while inside there is an abundance of *Ureibacillus chungkukjangi*. At the end of the cave, the greatest diversity is found, with 13 identified species, among which the most abundant are *Streptomyces glycanivorans*, *Paucisalibacillus globulus*, and *Planococcus liqunii*.

By comparing the identifications of the cultivable fraction obtained in Covadura and C3 caves, only three species are common to both caves: *Micrococcus luteus*, *Terribacillus* sp., and *Peribacillus frigoritolerans.* This last species was found both inside and outside the two caves. *Bacillus atrophaeus* was collected outside Covadura and inside C3 caves.

### Non-cultivable Bacteria

The NGS data yield very different results when compared with those of cultivable bacteria. This methodology was able to identify 749 genera of bacteria from 1889 ASVs clustered at the species level within the two caves.

#### Diversity Analysis

**Fig. 4:**
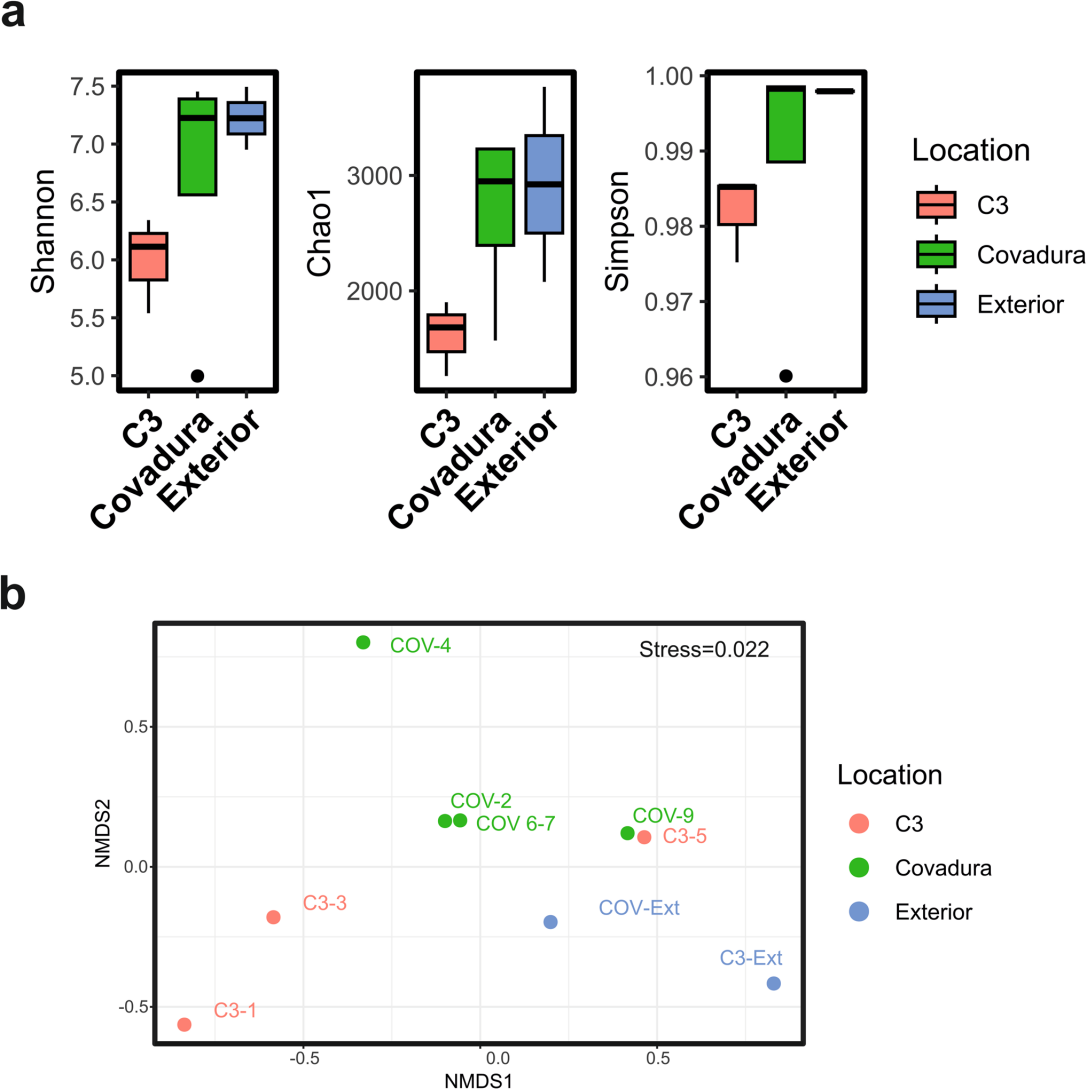
Diversity analysis. **a** Boxplot showing Shannon–Wiener, Chao1, and Simpson indices in C3, Covadura, and exterior samples. **b** Non-metric multidimensional scaling (NMDS) ordination. NMDS plot showing the spatial relationships among microbial communities from C3, Covadura, and exterior samples. The stress value of 0.022 indicates a good fit of the ordination to the original dissimilarity matrix

Covadura Cave showed the highest Shannon diversity, with a median value around 7.37, followed by the exterior and C3 samples with median values around 6.95 and 6.11, respectively (Fig. 4a and Table S4). In Covadura, there is an outlier point corresponding to the end of the lower gallery (COV-4). The Shannon–Wiener, Simpson, and Chao1 indices did not differ significantly between caves (Shannon: *F* = 1.268, *p* = 0.347; Simpson: *χ*^2^ = 2.5, *p* = 0.287; Chao1: *F* = 2.322, *p* = 0.179). However, the boxplot shows higher alpha diversity in Covadura and exterior samples compared to C3. This lower diversity in C3 is likely due to the cave’s smaller size and greater environmental stability, which may limit the variety of microbial niches.

Beta diversity was visualized by non-metric multidimensional scaling (NMDS) using the Bray–Curtis distance to show the dissimilarity between bacterial communities (Fig. 4b). NMDS analysis depicts Covadura samples separated from a cluster of C3 samples in the lower-left quadrant and exterior samples in the lower-right quadrant, suggesting a relatively distinct community composition. One sample from Covadura (COV-4) appears separated from the other samples. Some samples from deeper locations of both caves (COV-4 and C3-5) showed overlap.

#### Relative Abundances

**Fig. 5:**
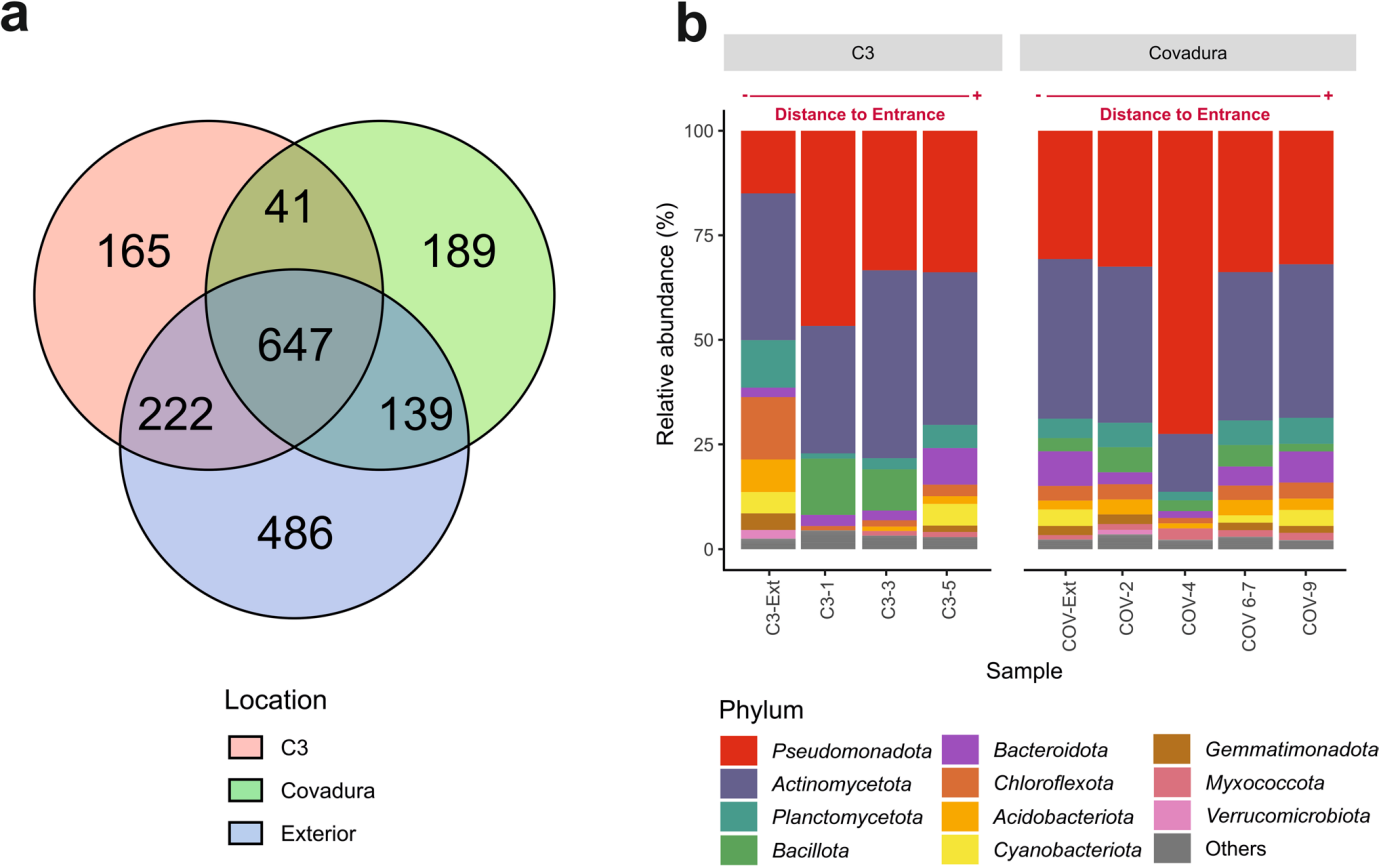
**a** Venn diagram showing the number of species unique to and shared among Covadura, C3, and exterior samples. **b** Bar plot shows the relative abundance of bacterial phyla in C3, Covadura, and exterior samples. The distance from the entrance of each sample is indicated at the top of the bar plot. “Others” groups the bacterial phyla with relative abundances below 1% in all samples

Figure 5a shows a high percentage of shared species across all samples (647 species, 34%), suggesting the occurrence of a common core in the airborne bacterial community. More unique species were found in the external air samples (486 species, 26%) compared to the cave samples (C3: 165 species, 9%; Covadura: 189 species, 10%). The lowest number of shared species was observed in the overlap between C3 and Covadura (41 species, 2%).

*Halococcus* (*Archaea*) was only represented in three cave samples, with percentages below 0.20% in Covadura (COV-2) and C3 (C3-1, C3-3); the rest of ASVs correspond to *Bacteria*. Figure 5b shows the most abundant ASVs classified at the phylum level. Of a total of 33 bacterial phyla identified, only 11 showed abundances above 1%. All samples were dominated by the phyla Pseudomonadota (14.98–72.52%) and Actinomycetota (13.75–44.88%). Other major bacterial phyla were Chloroflexota (1.04–14.84%), Planctomycetota (1.26–11.37%), Bacillota (0.20–13.46%), Bacteroidota (1.65–8.77%), Acidobacteriota (0.58–7.80%), and Cyanobacteriota (0.29–5.19%). Although the sample COV-4 shows a high percentage of Pseudomonadota compared to the rest of the samples, the Covadura samples show a high similarity in the percentage distribution of phyla, even with the exterior sample. In contrast, C3 samples display greater variability in phylum composition between locations. In the external air of C3 Cave, Planctomycetota, Chloroflexota, and Acidobacteriota appeared with abundances (> 5%), higher than in the rest of the samples.

It is important to highlight the occurrence of higher relative abundances of phototrophic taxa like Cyanobacteria not only in external air samples (C3-Ext and COV-Ext) but also in deeper locations in cave (C3-5, COV 6–7, and COV-9).

**Fig. 6:**
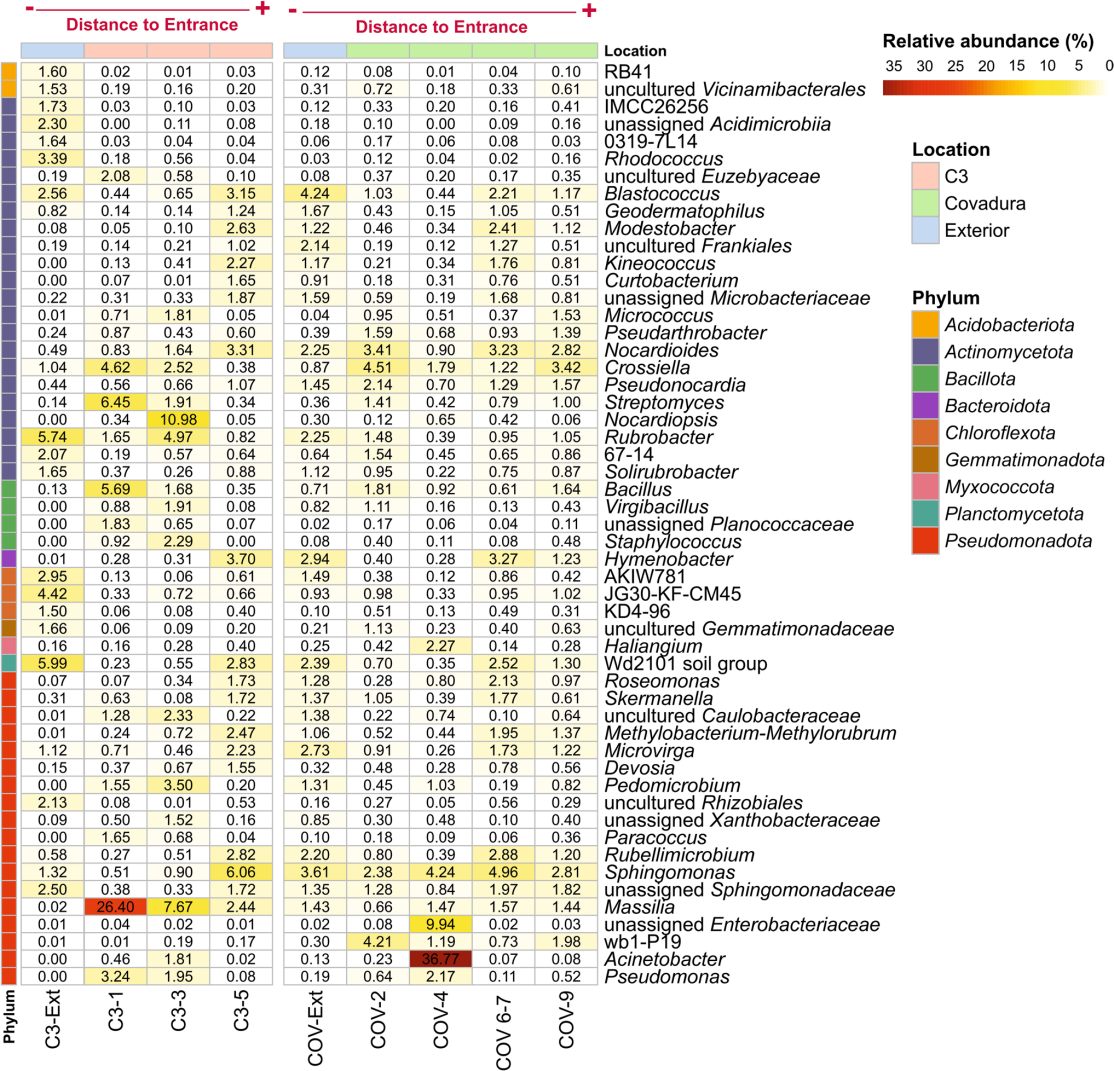
Heatmap showing the most abundant genera in the air samples of C3, Covadura, and exterior. Only genera with relative abundances above 1.5% in at least one sample are shown. The location of each sample and its distance from the cave entrance are indicated at the top of the heatmap

Figure 6 provides a visual representation of the most abundant ASVs classified at the genus level. The abundances of *Acinetobacter* and Enterobacteriaceae in Covadura Cave are notable, particularly in the sample COV-4, where they reached 36.77% and 9.94%, respectively. In C3 Cave, *Massilia* was highly abundant in the cave sample near the entrance (C3-1, 26.40%), and its relative abundance progressively decreased toward the interior, with 7.67% in C3-3 and 2.44% in C3-5. *Nocardiopsis* showed a high abundance in C3-3 (10.98%) but remained below 1% in the rest of the samples. Also notable are the abundances of *Rubrobacter*, *Streptomyces*, and *Bacillus* in C3 Cave (> 5%). In C3 Cave, similar decreasing trends from the ecotone area toward the inner cave locations, although less pronounced, were observed for *Streptomyces* and *Bacillus*.

With relative abundance > 2%, widespread genera retrieved in the air of different locations included *Rubrobacter*, *Crossiella*, *Blastococcus*, *Rubellimicrobium*, *Hymenobacter*, *Pseudomonas*, and WD2101 soil group*.* In Covadura Cave, the presence of the genera wb1-P19 and *Sphingomonas* is also remarkable*.* In C3 Cave, some of these genera also showed clear spatial trends, although less pronounced, where the relative abundance progressively decreased from the cave entrance to the interior. This pattern was observed for *Streptomyces* (4.62% → 2.56% → 0.67%), *Crossiella* (4.51% → 1.79% → 1.22%), *Bacillus* (1.04% → 0.87% → 0.65%), and Euzebyaceae (2.27% → 1.14% → 0.87%). In contrast, genera such as *Sphingomonas*, *Blastococcus*, *Nocardioides*, *Methylobacterium*–*Methylorubrum*, and wb1-P19 showed a slight pattern of increasing relative abundances toward the deeper zones. In external air, AKIW781 showed a notably higher relative abundance (> 1%), particularly in C3-Ext (2.95%), compared to its low percentages inside the caves. In the external air of Covadura Cave, *Sphingomonas*, *Nocardioides*, uncultured *Frankiales*, *Microvirga*, and *Rubellimicrobium* were observed with abundances (> 2%), and in C3 Cave, *Rhodococcus, Solirubrobacterales* genus 67–14, unassigned *Acidomicrobiia*, *Chloroflexota* JG30-KF-CM45 and AKIW781, and uncultured *Rhizobiales*. It is worth mentioning that the occurrence of interesting genera such as *Microvirga*, *Blastococcus*, and *Rubellimicrobium* showed a slight tendency pattern characterized by higher relative abundances in external air as well as in deeper cave locations.

#### Cultivable Versus Non-cultivable Bacteria

**Fig. 7:**
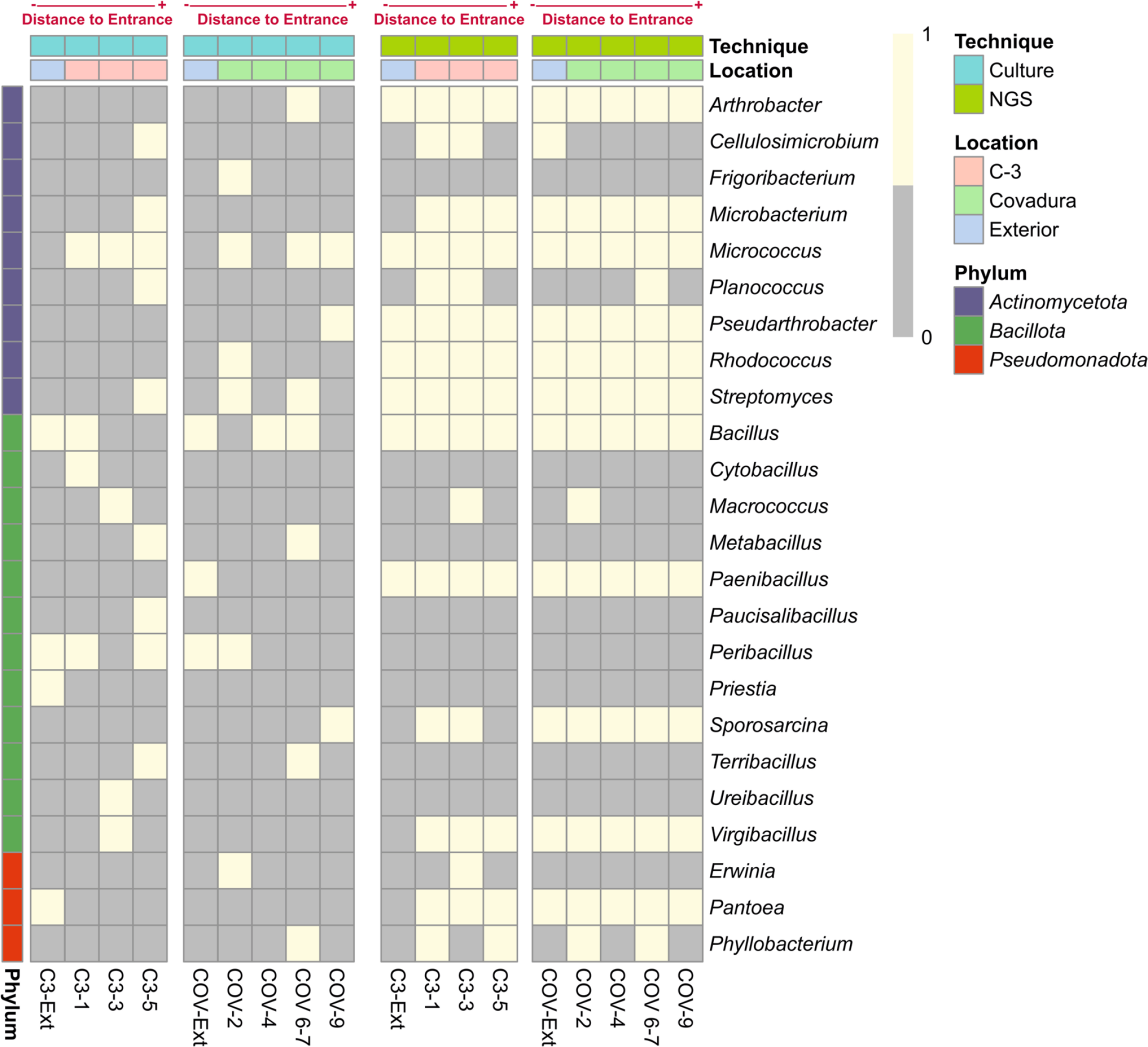
Presence-absence map to compare bacterial genera detected by culture and NGS techniques. Genera are listed on the y-axis, and samples are grouped by location (C3, Covadura, and exterior) and technique (NGS, culture). The location of each sample and its distance from the cave entrance are indicated at the top of the plot

Figure 7 compares the cultivable bacteria at the genus level with those detected by NGS. With relative abundances > 1% in the NGS dataset, the genera *Streptomyces*, *Bacillus*, *Rhodococcus*, *Arthrobacter*, *Pseudoarthrobacter*, *Micrococcus*, and *Virgibacillus* were also retrieved among the cultivable isolates. Of these, only the genera *Streptomyces*, *Bacillus*, and *Micrococcus* were isolated from the air of the two caves. Other genera such as *Microbacterium*, *Cellulosimicrobium*, *Planococcus*, *Macrococcus*, *Sporosarcina*, *Erwinia*, *Pantoea*, *Phyllobacterium*, and *Paenibacillus* with abundances < 1% in NGS were recovered through culture. Of the 24 cultivable genera, most of them were also identified by NGS in more locations compared to the culture technique. Only eight cultivable genera (*Ureibacillus*, *Frigoribacterium*, *Cytobacillus*, *Priestia*, *Peribacillus*, *Metabacillus*, *Paucisalibacillus*, and *Terribacillu*s) were not found in NGS. *Erwinia* was only detected in sample C3-3 by NGS and was isolated by culture only in COV-2.

To assess whether these genera were absent from the NGS dataset due to discrepancies between the NCBI and SILVA 138 databases, naive Bayes classifiers were trained in QIIME for the primer set 616F–1510R used for the culture approach, against the SILVA 138 database (Tables S5, S6). This classifier was used to classify the sequences obtained from cultured isolates. The results showed that *Peribacillus*, *Cytobacillus*, *Metabacillus*, and *Priestia* do not have any reference sequences registered in SILVA 138. Therefore, the species *Metabacillus schmidteae*, *Cytobacillus oceanisediminis*, *Priestia filamentosa*, and *Peribacillus frigoritolerans* were all classified under the genus *Bacillus*. Although the reference sequences of *Ureibacillus*, *Paucisalibacillus*, and *Frigoribacterium* are present in the SILVA database, they were not correctly classified. For example, *U. chungkukjangi* was classified as *Lysinibacillus* and *Erwinia endophytica* as *Lelliottia*. Furthermore, the genera *Terribacillus*, *Paucisalibacillus*, and *Frigoribacterium* were correctly identified at the genus level by the SILVA-trained classifier, but none of them was detected in any sample in the NGS dataset.

## Discussion

### Monitoring of Microclimate and Cave Air Renewal

The gases CH_4_ and CO_2_ are usually used as tracers for ventilation studies in caves because their concentrations depend largely on microclimatic variations within the cave and on the mass and energy transfer between the cave, epikarst, and soils, all of which are controlled by external climatic changes [3, 20, 29]. In particular, the variation in the abundance of CH_4_ [18, 30] and the relationship between CH_4_ and δ^13^C-CH_4_ in cave air [20] are key parameters for assessing the ventilation of subterranean atmospheres. Comparison of CO_2_ and CH_4_ enables an assessment of the influence of the exterior atmosphere and soil on the gas content of cave air, that is, the degree of isolation of the subsurface. In fact, lower levels of CH_4_ with ^12^C depletion attributed to methanotrophic oxidation in cave sediments are observed in the most isolated cave zones [22], and low CO_2_ levels may indicate either enhanced ventilation through exchange with the outside atmosphere or a reduced contribution of CO_2_ derived from soil. This CH_4_ consumption aligns with the distribution of methanotrophic taxa detected in the air samples, such as *Methylobacterium-Methylorubrum* [31], which were more abundant in the most isolated areas. On the other hand, another possible methanotroph, wb1-P19 [32], was highly abundant in the most ventilated cave, Covadura.

The inverse relationship between CH_4_ and CO_2_ concentrations reflects differences in ventilation dynamics between the C3 and Covadura caves. Based on these results, the three ventilation subzones identified in Covadura (Fig. 1B) are the result of prevailing gas exchange through the cave entrance and other discontinuities in the gypsum stratum overlying the upper galleries. This morphology results in a gradient characterized by a progressive increase in CH_4_ concentration and a decrease in CO_2_ levels toward the cave’s upper reaches, reflecting the differential dynamics of gas exchange along the cave profile.

Although both caves are well-mixed with the outer atmosphere throughout the year, the air renewal substantially increases during winter, when the colder and denser air from exterior inlets enters the subterranean environments. This air mixing between the caves and the exterior is supported by the high number of shared ASVs in all air samples, as shown in the Venn diagram (Fig. 5a). These results indicate that a part of the cave air microbiome originates from the connection of both caves with the exterior atmosphere. Likewise, a higher proportion of air enriched with atmospheric CH_4_ is progressively transferred from the open atmosphere to the caves, particularly in the most surficial galleries with wide connections with the exterior, such as the upper gallery of Covadura Cave. The higher CH_4_ depletion with narrow increases in CO_2_ indicates a dominant influx of outer air into both caves, with an almost null contribution of soil-derived CO_2_, and also a noteworthy CH_4_ consumption that increases in the most isolated locations, such as C3 Cave and inner locations of the lower gallery of Covadura Cave. Only in these most isolated locations, CO_2_ diffusion from the soil is slightly noted over the prevailing influx of exterior air with the atmospheric CH_4_ levels and lower CO_2_ concentrations.

Although ventilation patterns were well defined by CH_4_ and CO_2_ gas tracers, no clear patterns were found in the distribution of airborne bacterial populations across samples in the SAS study. In the culture technique, *Peribacillus* appears both in the exterior of Covadura and in COV-2, which could be related to ventilation. The fact that the NGS samples of Covadura show high similarity in distribution at the phylum level with the exterior atmosphere could reflect its more ventilated dynamics compared to C3 Cave. However, some abundant taxa show distribution patterns against the ventilation pattern which reveal complex ecological interactions. For example, phototrophic *Cyanobacteria*, which would be expected to decrease toward the less ventilated inner zones of the caves, appear with abundances similar to those in the exterior atmosphere. Other taxa such as *Microvirga*, *Blastococcus*, and *Rubellimicrobium* also showed this pattern, characterized by higher relative abundances in the external air but also in deeper cave locations.

Previous studies using culturable aerobiology techniques and microclimate monitoring in stratified caves like Covadura have shown that ventilation conditions influence the spatial heterogeneity of the airborne microbiota [18], although the authors also highlight that areas of a cave with a more active aerodynamic regime would experience significantly less impact from visitors and soil particle movement compared to areas with greater environmental stability [8]. This could explain the pattern observed in the isolated areas of the caves from the Gypsum Karst of Sorbas, especially considering that the SUPER 360 and the Coriolis air samplers had to be started manually. Other authors have reported this issue and established the main strategy used in this study, leaving the sampling site after turning on the device, as standard practice [33]. However, at least in caves, this standard practice could be insufficient since the disturbance of sediment particles in isolated zones could mask the influence of ventilation patterns in the microbial communities.

### Cultivable Airborne Bacteria

For most authors, there is a general consensus on the complementarity of data provided by the study of cultivable and non-cultivable airborne microorganisms. However, studies based on the cultivation of microorganisms have some limitations since those non-cultivable under specific laboratory conditions remain undetected, despite their abundance, and this fact underestimates the diversity of aerobiomes [34, 35]. Furthermore, there may also be an overrepresentation of spore-forming bacteria, which may not correspond to their actual abundance [36].

The cultivation and identification of microorganisms present in the atmosphere have shown that Gram-positive bacteria forming spores (*Bacillus* and other related genera within the Bacillota phylum, as well as the Actinomycetota, *Micrococcus*, and *Streptomyces*) are usually the most abundant groups, while the occurrence of Gram-negative bacteria is much lower [34]. This pattern is also mimicked in the air of caves [7]. However, this trend is not reflected in the NGS library (Figs. 6 and 7), which shows an unexpected abundance and diversity of airborne Gram-negative bacteria [36, 37], which were not found among the cultivable bacteria in the air of the gypsum caves.

When comparing the cultivable bacteria present in the air of the gypsum caves with those of other Andalusian limestone caves (Gruta de las Maravillas, Ardales, and Tesoro), a common pattern was observed in all these caves: the abundant occurrence of *Micrococcus luteus* [7]. Other abundant genera were *Pseudoarthrobacter siccitolerans* and *Arthrobacter* spp. in Ardales Cave, as well as *Micrococcus endophyticus* in Tesoro and Gruta de las Maravillas caves [7]. In the limestone caves, the low abundance and diversity of species of *Bacillus* and related genera, which are so abundant in the gypsum caves, were notable. This is probably related to the lower relative humidity in the gypsum caves, located in the semi-arid Sorbas region, and the production of endospores by the bacteria that inhabit the soils of the region and these caves.

### Non-cultivable Airborne Bacteria

NGS analysis revealed a high diversity of airborne bacterial taxa in both caves, with higher diversity values in Covadura Cave. The high occurrence of *Acinetobacter*, uncultured Enterobacteriaceae, and *Sphingomonas* in the sample COV-4 of Covadura is remarkable, as well as their low or insignificant abundance in C3 Cave, which is likely related to this particular sampling site (Fig. 7). This sample also showed an exceptionally spatially microbial distance from other Covadura samples and also very different from other samples in the culture approach, as it only showed cultivable *Bacillus*, while other sampling points included *Micrococcus*, *Peribacillus*, *Arthrobacter*, and *Erwinia*, among the most abundant cultivable bacteria. Together, these results suggest that COV-4 represents a unique microbiological hotspot shaped by site-specific environmental factors. In fact, COV-4 site in Covadura Cave is characterized by water condensation on the ceiling and a common contribution of organic matter in the area, due to a direct connection with the exterior through a parallel gallery that ends in a vertical shaft at the base of the access doline to the upper galleries (Fig. 1B). In this parallel gallery, there are usually stagnant waters that can accumulate dung from cattle or small animals that fall through the well of that gallery.

*Acinetobacter*, uncultured Enterobacteriaceae, and *Sphingomonas* are usually retrieved in caves. Members of these families and genera were frequently found in the air of caves subjected to visitor impacts [4, 8], cave pools, and flowing streams [38], bat microbiota, and guano [39]. The particular environmental characteristics of COV-4 sampling location in Covadura Cave (stagnant waters and accumulation of organic matter from the exterior, etc.) support the abundance of these three phylotypes.

Some of the most abundant genera identified in the present study (*Acinetobacter*, *Bacillus*, *Crossiella*, *Massilia*, *Pseudomonas*, *Rhodococcus*, *Rubrobacter*, etc.) have previously been included among the 20 most abundant bacterial phylotypes in caves [40]. *Massilia*, *Rubrobacter*, and *Bacillus* were relatively abundant in C3 Cave, in addition to *Nocardiopsis*, *Streptomyces*, and WD2101_soil_group. The high occurrence of Actinomycetota (*Rubrobacter*, *Nocardiopsis*, *Streptomyces*) in C3 Cave is consistent with other cave reports that describe this phylum as one of the most abundant in caves [41–43]. *Massilia* is ubiquitous in the environment, with more than 60 validly described species isolated from soils, water, air, rocks, and human clinical samples [7, 44, 45]. The genera *Rubrobacter* and WD2101_soil_group, with abundances greater than 5%, were retrieved in the sampling carried out in the exterior air of C3 Cave. This is not surprising given the abundances of both genera in arid soils [46, 47], such as those found in Sorbas. Other remarkable air gypsum cave bacteria retrieved in the NGS library that are also frequently recovered in arid environments and deserts were *Acinetobacter*, *Massilia*, *Blastococcus*, *Geodermatophilus*, *Streptomyces*, *Crossiella*, *Hymenobacter*, and *Sphingomonas*. Many of these genera are recognized components of the core microbiome of soils and airborne dust in arid environments, including the nearby Tabernas Desert [48, 49]. Their consistent occurrence in cave samples suggests that local edaphic inputs and atmospheric transport can also play an important role in the gypsum cave aerobiome.

Together, these results point to the fact that the origin of aerobiomes from gypsum caves responds to complex ecological interactions. The environmental conditions of Covadura and C3 caves are very different, although from a geological perspective, they have a common origin and are very close in location. Perhaps this is the reason why they share fewer species (41) in the Venn diagram (Fig. 5) than between each one and the exterior (222 for C3 and 139 for Covadura).

C3 is a small, stable cavity with a temperature around 17 °C and moderate humidity levels (88–92%), with a high degree of isolation from the exterior at the time of sampling. This homogeneity is reflected in lower diversity indices (Fig. 4a). The NGS results show a fairly clear pattern in the distribution of several genera. Thus, *Massilia* is very high in the cave area near the entrance and clearly decreases toward the interior. The same pattern, although less pronounced, appears for *Streptomyces*, *Crossiella*, *Bacillus*, and Euzebyaceae, among others. An opposite pattern is seen in *Sphingomonas*, *Blastococcus*, *Nocardioides*, and *Kineococcus*, and some potential methanotrophic genera that increase inwards.

Covadura Cave presents very different environmental conditions from C3 Cave. The temperature is much lower since the cavity behaves like a cold air trap, especially pronounced in the interior areas, which are 5 °C lower than the average temperature of C3. Furthermore, three distinct bioclimatic zones are observed, one of which, COV-4, has very special and distinctive conditions (which coincides with a completely different microbiological composition of the air; see Fig. 5b). At this point, the predominant presence of *Acinetobacter* and Enterobacteriaceae suggests a higher availability of organic matter, as described above.

COV-9 zone in Covadura Cave also displays a distinctive composition, representing an internal zone partially disconnected from the rest of the cave, where abundance patterns are altered. For example, the proportions of *Crossiella*, *Bacillus*, and Euzebyaceae follow a pattern of declining from COV-2 zone to COV-4 and COV 6–7 zones, similar to that observed in C3, but altered in COV-9 zone.

A previous NGS study on the yellow biofilms present in the walls of Covadura and C3 caves was published [6], and an evident similarity was found between the biofilm genera and those collected from the air, as reported here. In the biofilms covering the walls of these two caves, the most abundant genera were *Crossiella*, the non-cultivable genera wb1-P19, the non-cultivable Euzebyaceae, the Actinomycetota 0319–7134, and the Pseudomonadota Ga0077536. Remarkably, the three most abundant genera in the biofilms were also retrieved in the air. A plausible explanation for the similarities between the genera found in the air and in the cave biofilms was revealed by Martin-Pozas et al. [17] in their aerobiology research in a limestone cave. These authors reported that the most abundant bacteria in the cave biofilms also appeared in the sediments and suggested that the cave sediments can act as bacterial reservoirs that can be mobilized by airborne particles through air currents or human visitors. Other cave studies based on culture approaches reported that the most abundant species in the air of La Garma Cave (northern Spain): *Streptomyces cyaneofuscatus*, *Streptomyces pratensis*, *Streptomyces avidinii*, *Peribacillus simplex*, *Bacillus licheniformis*, *Rhodococcus erythropolis*, and *Stenotrophomonas maltophilia* were also isolated from the biofilms [8]. These authors concluded that the bacteria from the cave biofilms and sediments were released into the air by anthropogenic activities such as archaeological excavations, footsteps of visitors that removed the biofilms, air currents, and animals present in the cave.

These findings support the idea that the cave air microbiota in the caves from the Gypsum Karst of Sorbas has a complex origin, shaped by internal sources such as sediments, biofilms, and guano, as well as by external inputs of organic matter from surrounding arid environments and atmospheric transport.

### Cultivable Versus Non-cultivable Bacteria

Most of the genera from the NGS dataset are difficult to cultivate in standard media, and only a few of them were recovered using the SAS methodology, which denotes the bias introduced with this approach. When comparing the bacteria identified with SAS and NGS, it is noticed that most of the species isolated in Covadura and C3 caves were not represented among the species of the NGS library. Some of them could not be correctly identified with the NGS approach. This is not surprising because the identification of isolates is carried out with ~ 900 bp and the identification of species with Illumina is done with ~ 430 bp. Therefore, the identification of bacteria is more accurate with SAS than with Short Amplicon Sequencing, and this can explain the discrepancy in identification captured by both approaches.

Although some authors reported a different shift in microbiomes when comparing culture-dependent and -independent methods [50–52], there is some correspondence between both methods, as only 16 of 24 SAS genera were represented in the NGS library. Thus, six of these genera presented relative abundances > 1% (*Micrococcus*, *Pseudarthrobacter*, *Bacillus*, *Streptomyces*, *Arthrobacter*, and *Virgibacillus*); ten appeared with relative abundances < 0.5% (*Rhodococcus*, *Sporosarcina*, *Phyllobacterium*, *Erwinia*, *Macrococcus*, *Microbacterium*, *Cellulosimicrobium*, *Planococcus*, *Paenibacillus*, and *Pantoea*); four were classified under the genus *Bacillus* (*Peribacillus*, *Cytobacillus*, *Metabacillus*, and *Priestia*), and three were absent in the NGS (*Terribacillus*, *Frigoribacterium*, and *Paucibacillus*). Some species such as *Phyllobacterium myrsinacearum*, *Erwinia endophytica*, and *Ureibacillus chungkukjangi* could not be correctly classified at the genus level with the NGS approach.

Most identified cultivable bacteria are included in the group of endospore-forming bacteria, and there is evidence of the difficulty of extracting DNA from these spores [53]. Other authors indicate that the type of kit used for DNA extraction conditions the results of the analyses [54]. It is reasonable to expect that the samples obtained for the NGS study will contain endospore-forming bacteria and that they could be resistant to many of the traditional DNA extraction methods and, therefore, potentially undetectable. Filippidou et al. [55] pointed out that not all microbial species react in the same way to DNA extraction, mainly due to the diversity of morphological and physiological states of environmental bacteria, and that therefore Bacillota (endospore-forming bacteria) are underrepresented in metagenomic libraries. However, in this study, a large number of sequences related to Bacillota were obtained with NGS, sometimes even a greater number of genera compared to the culture approach. For example, *Bacillus* was isolated from only five samples using the culture approach, while NGS detected sequences in all samples. A similar pattern was observed with the genus *Paenibacillus*. In addition, Jurado et al. [56], using the same DNA extraction methodology as here, studied the microbial communities of phototrophic biofilms on speleothems from Nerja Cave, Spain. The relative abundances of *Bacillus* in all samples ranged between 36.5 and 63.2%, which demonstrated that *Bacillus* can be retrieved with the DNA extraction methodology used here.

Despite the differences detected in the results obtained by the SAS and NGS approaches, is the use of SAS useful in aerobiology? Some previous studies suggest that SAS allows the isolation and identification of several bacteria not included in the NGS library, and also of new species of bacteria not yet described [57], which allows the study of their biotechnological potential [58, 59]. Nevertheless, the results of this study clearly show that although the culture-based approach provides more reliable species-level classification, it captures significantly less microbial diversity. Most genera isolated by SAS were also detected in the NGS dataset, in low relative abundances or under different taxonomic assignments due to database and primer limitations. A possible solution to the problems caused by the divergent identification of bacteria using SAS and NGS could be the profiling of the air microbiome through long-read sequencing technologies, which can resolve species-level annotations and specific ecosystem functions through de novo metagenomic assemblies, as reported by Reska et al. [60]. In addition, functional annotation to the metagenomic dataset could allow one to assess the presence of general metabolic pathways and ecosystem functions. Therefore, although SAS undoubtedly plays an important role in isolating new species with potential biotechnological applications, it does not appear to provide significant added value for airborne environmental microbiology studies in caves.

## Conclusions

NGS and metagenomics have revolutionized aerobiome research by enabling comprehensive analyses of microbial diversity and functionality, far beyond the capabilities of traditional culture-based methods such as SAS. This study highlights a marked divergence between data obtained through cultivable (SAS) and non-cultivable (NGS) approaches. Cultivable genera and species, particularly spore-forming bacteria from the phyla Bacillota (e.g., *Bacillus*) and Actinomycetota (e.g., *Micrococcus*, *Streptomyces*), were overrepresented in SAS results but were either absent or present at low relative abundances in NGS datasets. This reflects a strong bias toward Gram-positive, spore-forming bacteria that readily grow on culture media but do not accurately represent the airborne microbial community.

NGS revealed a much broader spectrum of airborne bacterial genera in Covadura and C3 caves, many of which are typically associated with desert and arid soils, as those found in the Gypsum Karst of Sorbas. Notably, several of the most abundant bacteria found in cave biofilms and sediments were only detected in air samples through NGS, suggesting that a portion of the airborne microbiota originates from cave-associated microbial communities. These communities appear to be shaped by environmental factors such as ventilation dynamics and air stagnation, which define distinct microhabitats within each cave. Such ecological patterns were not captured by culture-based methods, which failed to provide meaningful spatial or functional insights.

Furthermore, NGS data revealed a dual origin for cave airborne bacteria—both from internal cave sources and external air—whereas SAS results were limited to detecting similar Gram-positive bacteria inside and outside the caves, which provides little ecological resolution. This culture bias has previously been documented in other cave studies.

Environmental measurements, including CH_4_ and CO_2_ gas tracers, effectively identified ventilation and stagnation zones within the caves. These zones corresponded to distinct microbial distribution patterns detected by NGS-based aerobiological analysis, reinforcing the link between cave microclimate and airborne microbial ecology.

In conclusion, NGS provides a more accurate and ecologically meaningful representation of the cave aerobiome, capturing both diversity and spatial dynamics. In contrast, SAS and culturing methods select a narrow subset of bacteria, leading to biased and incomplete interpretations. To obtain reliable results in aerobiological studies of subterranean environments, it is essential to integrate high-throughput sequencing techniques with detailed environmental monitoring—including temperature, humidity, and gas tracer concentrations—to fully understand the origin, distribution, and ecological roles of airborne microorganisms.

## Supplementary Information

Below is the link to the electronic supplementary material.

Supplementary file1 (DOCX 321 KB)

## Data Availability

The 16S rRNA gene sequences and accompanying metadata from this study were deposited in the Sequence Read Archive (SRA) of NCBI under the project number PRJNA1237874. Accession numbers of the bacteria were PV334950-PV334999.
