# Supplementary material for: Aerobiology and Environmental Zonation in Gypsum Caves: A Comparative Study of Culturing and NGS Approaches
Source: Microb Ecol. 2025 Sep 30;88:95. doi: 10.1007/s00248-025-02591-4 (PMC12484262; doi:10.1007/s00248-025-02591-4)
Supplement: Supplementary file 1 [file 248_2025_2591_MOESM1_ESM.docx]

**SUPPLEMENTARY INFORMATION**

**Aerobiology and Environmental Zonation in Gypsum Caves: A Comparative Study of Culturing and NGS Approaches**

Tamara Martin-Pozas^1^, Angel Fernandez-Cortes^2^, Jose Maria Calaforra^2^, Sergio Sanchez-Moral^3^, Cesareo Saiz-Jimenez^1^, Valme Jurado^1^

^1^Instituto de Recursos Naturales y Agrobiologia de Sevilla, IRNAS-CSIC, 41012 Sevilla, Spain. v.jurado@csic.es; tmpozas@csic.es; saiz@irnase.csic.es

^2^Departamento de Biologia y Geologia, Universidad de Almeria, 04120Almeria, Spain.

acortes@ual.es; jmcalaforra@ual.es

^3^Museo Nacional de Ciencias Naturales, MNCN-CSIC, 28006 Madrid, Spain.

ssmilk@mncn.csic.es

Corresponding author: Cesareo Saiz-Jimenez: saiz@irnase.csic.es

**Figure S1.** Views of galleries and dolines to access the Covadura and C3 caves. a: Collapse doline to access the upper gallery of Covadura Cave (the vertical yellow arrow indicates the human-made wellhead that directly connects through a parallel gallery with the main gallery at the second level (photo b). b: Beginning of the main gallery at the second level of Covadura Cave (sampling point COV-4 in Table S1 and Figure 1A). The yellow arrow indicates the connection with the exterior through a parallel gallery that ends at the base of the well displayed in photo a). c: Vertical shaft connecting the upper gallery with the main lower gallery in Covadura Cave (between the sampling points COV-3 and COV-4; Table S1 and Figure 1A). D: Doline to access C3 Cave partially blocked by a fig tree. e: Detailed view of a characteristic narrow section of C3 Cave (sampling point C3-3 in Table S1 and Figure 1B). Cave photos by J. Les.

**Table S1. Location and description of aerobiological sampling.**

| **Gas** | **Cave** | **Location** | **T** | **HR** | **CO_2_** | **CH_4_** | **Distance to Entrance (m)** |
| --- | --- | --- | --- | --- | --- | --- | --- |
| C3-1 | C3 | Entrance  (Ecotone zone) | 16.60 | 89.3 | 588 | 0.36 | 33 |
| C3-3 | C3 | Intermediate (Isolated Zone) | 17.83 | 88.9 | 611 | 0.21 | 67 |
| C3-5 | C3 | Final  (Isolated Zone) | 17.25 | 92.1 | 533 | 0.22 | 117 |
| C3-Ext | Exterior C3 | Exterior | 29.56 | 34.4 | 425 | 2.00 | 3 m |
| COV-2 | Covadura | Intermediate Upper gallery  (Ecotone zone) | 17.97 | 94.7 | 468 | 1.96 | 33 |
| COV-4 | Covadura | End of lower gallery  (Intermediate Zone) | 14.09 | 93.7 | 454 | 1.79 | 73 |
| COV 6-7 | Covadura | Intermediate  Lower gallery  (Isolated Zone) | 12.58 | 91.7 | 452 | 1.57 | 176 |
| COV-9 | Covadura | Start of lower gallery  (Isolated Zone) | 12.09 | 78.9 | 521 | 1.19 | 256 |
| COV-Ext | Exterior Covadura | Exterior | 29.56 | 34.4 | 425 | 2.01 | 3 |

**Table S2.** Cultivable bacteria identified in the air of Covadura Cave

| **Covadura Cave** | **CFU/m^3*^** | **Abundance %** | **Identification NCBI** | **Accession Numbers** |
| --- | --- | --- | --- | --- |
| COV-9 | 40 ± 0 | 57.1 | *Micrococcus luteus* 99.76% | PV334950 |
|  |  | 28.6 | *Pseudarthrobacter psychrotolerans* 99.41% | PV334951 |
|  |  | 14.3 | *Sporosarcina globispora* 98.61% | PV334952 |
| COV 6-7 | 90 ± 80 | 38.8 | *Micrococcus luteus* 100% | PV334953 |
|  |  | 11.1 | *Bacillus* sp. (*B.* *haynesii/B. sonorensis*) 99.89% | PV334954 |
|  |  | 11.1 | *Phyllobacterium myrsinacearum* 97.91% | PV334955 |
|  |  | 5.6 | *Streptomyces* sp. (*S. praecox/S. cyaneofuscatus*) 99.88% | PV334956 |
|  |  | 5.6 | *Terribacillus* sp. (*T.* *saccharophilus/T. goriensis*) 99.66% | PV334957 |
|  |  | 22.2 | *Arthrobacter pascens* 99.88% | PV334958 |
|  |  | 5.6 | *Streptomyces goshikiensis* 99.88% | PV334959 |
| COV-4 | 20 ± 10 | 25.0 | [*Bacillus* sp. (*B. albus/B. wiedmannii*) 99.55%](https://www.ezbiocloud.net/taxonomy?tn=Bacillus%20toyonensis) | PV334960 |
|  |  | 50.0 | *Bacillus mojavensis* 99.77% | PV334961 |
|  |  | 25.0 | *Bacillus cereus* 100% | PV334962 |
| COV-2 | 70 ± 40 | 23.1 | *Erwinia endophytica* 98.01% | PV334963 |
|  |  | 30.8 | *Peribacillus frigoritolerans* 100% | PV334964 |
|  |  | 15.3 | *Micrococcus luteus* 100% | PV334965 |
|  |  | 7.7 | *Streptomyces rubiginosohelvolus* 100% | PV334966 |
|  |  | 15.4 | *Frigoribacterium endophyticum* 99.65% | PV334967 |
|  |  | 7.7 | *Rhodococcus erythropolis* 99.65% | PV334968 |
| COV-Ext | 70 ± 40 | 38.5 | *Bacillus mojavensis* 99.89% | PV334969 |
|  |  | 7.7 | *Paenibacillus lautus* 99.55% | PV334970 |
|  |  | 38.5 | *Peribacillus frigoritolerans* 100% | PV334971 |
|  |  | 15.3 | *Bacillus atrophaeus* 99.89% | PV334972 |

*The values correspond to the average of two replicates with standard errors. expressed as Colony Forming Units per m^3^ of sampled air.

**Table S3. Cultivable bacteria identified in the air of C3 Cave.**

| **C3 Cave** | **CFU/m^3*^** | **Abundance %** | **Identification NCBI** |  | **Accession numbers** |
| --- | --- | --- | --- | --- | --- |
| C3-1 | 260 ± 110 | 16.3 | *Bacillus atrophaeus* 100% | | PV334973 |
|  |  | 71.4 | *Peribacillus frigoritolerans*100% | | PV334974 |
|  |  | 4.1 | *Micrococcus luteus* 99.65% | | PV334975 |
|  |  | 4.1 | *Cytobacillus oceanisediminis* 99.77% | | PV334976 |
|  |  | 4.1 | *Micrococcus antarcticus* 99.65% |  | PV334977 |
| C3-3 | 50 ± 30 | 20.0 | *Micrococcus endophyticus* 99.76% | | PV334978 |
|  |  | 10.0 | *Macrococcus equipercicus* 99.66% | | PV334979 |
|  |  | 10.0 | *Virgibacillus jeotgali* 99.33% | | PV334980 |
|  |  | 50.0 | *Ureibacillus chungkukjangi* 99.89% | | PV334981 |
|  |  | 10.0 | *Micrococcus luteus* 99.76% | | PV334982 |
| C3-5 | 120 ± 30 | 8.3 | *Streptomyces exfoliatus* 99.76% | | PV334983 |
|  |  | 4.2 | *Metabacillus schmidteae* 98.34% | | PV334984 |
|  |  | 4.2 | *Micrococcus luteus* 99.88% | | PV334985 |
|  |  | 8.3 | *Micrococcus endophyticus* 99.88% | | PV334986 |
|  |  | 4.2 | *Terribacillus* sp. (*T.* *saccharophilus/T. goriensis*) 99.44% | | PV334987 |
|  |  | 16.7 | *Streptomyces glycanivorans* 99.65% | | PV334988 |
|  |  | 12.5 | *Paucisalibacillus globulus* 97.99% | | PV334989 |
|  |  | 4.2 | *Microbacterium flavescens* 98.82% | | PV334990 |
|  |  | 8.3 | *Microbacterium saccharophilum* 99.29% | | PV334991 |
|  |  | 12.4 | *Planococcus luteus* 99.28% | | PV334992 |
|  |  | 8.3 | *Peribacillus frigoritolerans*100% | | PV334993 |
|  |  | 4.2 | *Cellulosimicrobium funkei* 99.41% | | PV334994 |
|  |  | 4.2 | *Cellulosimicrobium cellulans* 99.88% | | PV334995 |
| C3-Ext | 180 ± 50 | 51.5 | *Peribacillus frigoritolerans*100% | | PV334996 |
|  |  | 39.4 | *Priestia filamentosa* 99.77% | | PV334997 |
|  |  | 6.1 | *Pantoea agglomerans* 99.62% | | PV334998 |
|  |  | 3.0 | *Bacillus pumilus*100% | | PV334999 |

*The values correspond to the average of two replicates with standard errors. expressed as Colony Forming Units per m^3^ of sampled air.

**Table S4. Alpha diversity results (NGS data)**

| **Samples** | **Location** | **Observed** | **Chao1** | **Shannon** | **Simpson** |
| --- | --- | --- | --- | --- | --- |
| C3-1 | C3 | 1261 | 1262 | 5.54 | 0.98 |
| C3-3 | C3 | 1677 | 1683 | 6.11 | 0.99 |
| C3-5 | C3 | 1899 | 1901 | 6.34 | 0.99 |
| C3-Ext | Exterior | 3751 | 3767 | 7.49 | 1.00 |
| COV-9 | Covadura | 2659 | 2669 | 7.08 | 1.00 |
| COV_6-7 | Covadura | 3222 | 3232 | 7.37 | 1.00 |
| COV-4 | Covadura | 1558 | 1570 | 5.00 | 0.96 |
| COV-2 | Covadura | 3225 | 3229 | 7.45 | 1.00 |
| COV-Ext | Exterior | 2075 | 2078 | 6.95 | 1.00 |

**Table S5. NCBI Identification of cultivable bacteria**

| **Sequence ID** | **NCBI Identification** | **Identity %** |
| --- | --- | --- |
| CV-1A.1-616F-0KVM5-def | [*Bacillus* sp. *(B. albus/B. wiedmannii)*](https://www.ezbiocloud.net/taxonomy?tn=Bacillus%20toyonensis) | 99.66 |
| CV-1A.2-616F-0KVM5-def | *Bacillus mojavensis* | 99.77 |
| CV-2B.1-616F-C9-0KVM5-def | *Bacillus cereus* | 100 |
| CV-6F.1-616F-0KVM5-def | *Micrococcus luteus* | 100 |
| CV-6F.2-616F-0KVM5-def | *Bacillus* sp. (*B.* *haynesii/B. sonorensis*) | 100 |
| CV-6F3-616F-0KVM5-def | *Phyllobacterium myrsinacearum* | 98.03 |
| CV-6F.5-616F-0KVM5-def | *Streptomyces* sp. (*S. praecox/S. cyaneofuscatus*) | 100 |
| CV-6F.6-616F-0KVM5-def | *Terribacillus* sp. (*T.* *saccharophilus/T. goriensis*) | 99.89 |
| CV-6F.7-616F-0KVM5-def | *Arthrobacter pascens* | 99.77 |
| CV-6F.8-616F-0KVM5-def | *Streptomyces goshikiensis* | 99.88 |
| CV-7G.1-616F-0KVM5-def | *Micrococcus luteus* | 99.88 |
| CV-7G.2-616F-0KVM5-def | *Pseudarthrobacter psychrotolerans* | 99.65 |
| CV-7G.3-616F-0KVM5-def | *Sporosarcina globispora* | 99.77 |
| CV-9I1-616F-0KVM5-def | *Erwinia endophytica* | 98.01 |
| CV-9I2-616F-0KVM5-def | *Peribacillus frigoritolerans* | 100 |
| CV-9I.3-616F-0KVM5-def | *Micrococcus luteus* | 100 |
| CV-9I.4-616F-0KVM5-def | *Streptomyces rubiginosohelvolus* | 99.88 |
| CV-9I.9-616F-0KVM5-def | *Frigoribacterium endophyticum* | 99.65 |
| CV-9I10-616F-0KVM5-def | *Rhodococcus erythropolis* | 99.88 |
| CV-12L1-616F-0KVM5-def | *Bacillus mojavensis* | 100 |
| CV-12L.2-616F-0KVM5-def | *Paenibacillus lautus* | 100 |
| CV-12L.3-616F-0KVM5-def | *Peribacillus frigoritolerans* | 100 |
| CV-12L.4-616F-0KVM5-def | *Bacillus atrophaeus* | 99.89 |
| C3-2B.1-616F-0KVM5-def | *Bacillus atrophaeus* | 100 |
| C3-2B.2-616F-0KVM5-def | *Peribacillus frigoritolerans* | 100 |
| C3-2B.3-616F-0KVM5-def | *Micrococcus luteus* | 99.76 |
| C3-2B.4-616F-0KVM5-def | *Cytobacillus oceanisediminis* | 99.89 |
| C3-2B6-6161F-0KVM5-def | *Micrococcus antarcticus* | 100 |
| C3-3C.1-616F-0KVM5-def | *Micrococcus endophyticus* | 99.88 |
| C3-3C.3-616F-0KVM5-def | *Macrococcus equipercicus* | 100 |
| C3-4D.1-616F-0KVM5-def | *Virgibacillus jeotgali* | 99.33 |
| C3-4D2-6161F-0KVM5-def | *Ureibacillus chungkukjangi* | 99.89 |
| C3-4D3-616F-0KVM5-def | *Micrococcus luteus* | 100 |
| C3-5E.1-616F-0KVM5-def | *Streptomyces exfoliatus* | 99.76 |
| C3-5E.2-616F-0KVM5-def | *Metabacillus schmidteae* | 98.7 |
| C3-5E.3-616F-0KVM5-def | *Micrococcus luteus* | 100 |
| C3-5E.4-616F-0KVM5-def | *Micrococcus endophyticus* | 100 |
| C3-5E5-6161F-0KVM5-def | *Terribacillus* sp. (*T.* *saccharophilus/T. goriensis*) | 99.89 |
| C3-5E6-616F-0KVM5-def | *Streptomyces glycanivorans* | 99.76 |
| C3-6F.1-616F-0KVM5-def | *Paucisalibacillus globulus* | 98.1 |
| C3-6F.2-616F-0KVM5-def | *Microbacterium flavescens* | 99.88 |
| C3-6F.3-616F-0KVM5-def | *Microbacterium saccharophilum* | 99.76 |
| C3-6F.4-616F-0KVM5-def | *Planococcus liqunii* | 99.76 |
| C3-6F.5-616F-0KVM5-def | *Peribacillus frigoritolerans* | 99.89 |
| C3-6F8-6161F-0KVM5-def | *Cellulosimicrobium funkei* | 99.88 |
| C3-6F9-616F-0KVM5-def | *Cellulosimicrobium cellulans* | 100 |
| C3-7G2-616F-0KVM5-def | *Peribacillus frigoritolerans* | 100 |
| C3-7G.3-616F-0KVM5-def | *Priestia filamentosa* | 100 |
| C3-8H2-6161F-0KVM5-def | *Pantoea agglomerans* | 99.75 |
| C3-8H3-616F-0KVM5-def | *Bacillus pumilus* | 100 |

**Table S6. SILVA Identification of cultivable bacteria.**

| **Sequence ID** | **SILVA Identification** | **Confidence** |
| --- | --- | --- |
| CV-1A.1-616F-0KVM5-def | *Bacillus* | 1.00 |
| CV-1A.2-616F-0KVM5-def | *Bacillus* | 1.00 |
| CV-2B.1-616F-C9-0KVM5-def | *Bacillus* | 1.00 |
| CV-6F.1-616F-0KVM5-def | *Micrococcus luteus* | 0.79 |
| CV-6F.2-616F-0KVM5-def | *Bacillus* | 1.00 |
| CV-6F3-616F-0KVM5-def | *Rhizobiaceae* | 1.00 |
| CV-6F.5-616F-0KVM5-def | *Streptomyces* | 1.00 |
| CV-6F.6-616F-0KVM5-def | *Terribacillus goriensis* | 1.00 |
| CV-6F.7-616F-0KVM5-def | *Micrococcaceae* | 1.00 |
| CV-6F.8-616F-0KVM5-def | *Streptomyces lavendulae* | 0.81 |
| CV-7G.1-616F-0KVM5-def | *Micrococcus luteus* | 0.80 |
| CV-7G.2-616F-0KVM5-def | *Micrococcaceae* | 1.00 |
| CV-7G.3-616F-0KVM5-def | *Sporosarcina globispora* | 0.70 |
| CV-9I1-616F-0KVM5-def | *Lelliottia;* s__bacterium_NXKED1 | 0.87 |
| CV-9I2-616F-0KVM5-def | *Bacillus* | 1.00 |
| CV-9I.3-616F-0KVM5-def | *Micrococcus_luteus* | 0.79 |
| CV-9I.4-616F-0KVM5-def | *Streptomyces* | 1.00 |
| CV-9I.9-616F-0KVM5-def | *Frigoribacterium endophyticum* | 0.80 |
| CV-9I10-616F-0KVM5-def | *Rhodococcus* | 1.00 |
| CV-12L1-616F-0KVM5-def | *Bacillus* | 1.00 |
| CV-12L.2-616F-0KVM5-def | *Paenibacillus lautus* | 0.91 |
| CV-12L.3-616F-0KVM5-def | *Bacillus* | 1.00 |
| CV-12L.4-616F-0KVM5-def | *Bacillus* | 1.00 |
| C3-2B.1-616F-0KVM5-def | *Bacillus* | 1.00 |
| C3-2B.2-616F-0KVM5-def | *Bacillus* | 1.00 |
| C3-2B.3-616F-0KVM5-def | *Micrococcus luteus* | 0.73 |
| C3-2B.4-616F-0KVM5-def | *Bacillus oceanisediminis* | 0.92 |
| C3-2B6-6161F-0KVM5-def | *Micrococcus* | 1.00 |
| C3-3C.1-616F-0KVM5-def | *Micrococcus* | 1.00 |
| C3-3C.3-616F-0KVM5-def | *Macrococcus equipercicus* | 0.82 |
| C3-4D.1-616F-0KVM5-def | *Bacillaceae* | 1.00 |
| C3-4D2-6161F-0KVM5-def | *Lysinibacillus* | 1.00 |
| C3-4D3-616F-0KVM5-def | *Micrococcus luteus* | 0.79 |
| C3-5E.1-616F-0KVM5-def | *Streptomyces venezuelae* | 0.75 |
| C3-5E.2-616F-0KVM5-def | *Bacillus* | 1.00 |
| C3-5E.3-616F-0KVM5-def | *Micrococcus luteus* | 0.79 |
| C3-5E.4-616F-0KVM5-def | *Micrococcus* | 1.00 |
| C3-5E5-6161F-0KVM5-def | *Terribacillus_goriensis* | 0.99 |
| C3-5E6-616F-0KVM5-def | *Streptomyces* | 1.00 |
| C3-6F.1-616F-0KVM5-def | *Paucisalibacillus;* s__uncultured_bacterium | 0.87 |
| C3-6F.2-616F-0KVM5-def | *Frigoribacterium* | 0.91 |
| C3-6F.3-616F-0KVM5-def | *Microbacterium* | 1.00 |
| C3-6F.4-616F-0KVM5-def | *Planomicrobium glaciei* | 0.97 |
| C3-6F.5-616F-0KVM5-def | *Bacillus* | 1.00 |
| C3-6F8-6161F-0KVM5-def | *Cellulosimicrobium cellulans* | 0.89 |
| C3-6F9-616F-0KVM5-def | *Cellulosimicrobium cellulans* | 0.77 |
| C3-7G2-616F-0KVM5-def | *Bacillus* | 1.00 |
| C3-7G.3-616F-0KVM5-def | *Bacillus filamentosus* | 1.00 |
| C3-8H2-6161F-0KVM5-def | *Pantoea* | 0.99 |
| C3-8H3-616F-0KVM5-def | *Bacillus* | 1.00 |
